# Intravenously delivered mesenchymal stem cell-derived exosomes target M2-type macrophages in the injured spinal cord

**DOI:** 10.1371/journal.pone.0190358

**Published:** 2018-01-02

**Authors:** Karen L. Lankford, Edgardo J. Arroyo, Katarzyna Nazimek, Krzysztof Bryniarski, Philip W. Askenase, Jeffery D. Kocsis

**Affiliations:** 1 Department of Neurology, Yale University School of Medicine, New Haven, Connecticut, United States of America; 2 Center for Neuroscience Regeneration Research, VA Connecticut Healthcare System, West Haven, Connecticut, United States of America; 3 Department of Immunology, Jagiellonian University College of Medicine, Krakow; Poland; 4 Section of Allergy and Clinical Immunology, Department of Internal Medicine, Yale University School of Medicine, New Haven, Connecticut, United States of America; Rutgers-Robert Wood Johnson Medical School, UNITED STATES

## Abstract

In a previous report we showed that intravenous infusion of bone marrow-derived mesenchymal stem cells (MSCs) improved functional recovery after contusive spinal cord injury (SCI) in the non-immunosuppressed rat, although the MSCs themselves were not detected at the spinal cord injury (SCI) site [1]. Rather, the MSCs lodged transiently in the lungs for about two days post-infusion. Preliminary studies and a recent report [2] suggest that the effects of intravenous (IV) infusion of MSCs could be mimicked by IV infusion of exosomes isolated from conditioned media of MSC cultures (MSC^exos^). In this study, we assessed the possible mechanism of MSC^exos^ action on SCI by investigating the tissue distribution and cellular targeting of DiR fluorescent labeled MSC^exos^ at 3 hours and 24 hours after IV infusion in rats with SCI. The IV delivered MSC^exos^ were detected in contused regions of the spinal cord, but not in the noninjured region of the spinal cord, and were also detected in the spleen, which was notably reduced in weight in the SCI rat, compared to control animals. DiR “hotspots” were specifically associated with CD206-expressing M2 macrophages in the spinal cord and this was confirmed by co-localization with anti-CD63 antibodies labeling a tetraspanin characteristically expressed on exosomes. Our findings that MSC^exos^ specifically target M2-type macrophages at the site of SCI, support the idea that extracellular vesicles, released by MSCs, may mediate at least some of the therapeutic effects of IV MSC administration.

## Introduction

Previous studies have shown that intravenous delivery of bone marrow derived mesenchymal stem cells (MSCs) can promote functional recovery in rodent models of contusive spinal cord injury (SCI) [1, 3–6], as well as accelerate the recovery of blood spinal cord barrier integrity [1]. Direct transplantation of MSCs into spinal cord lesions can reduce lesion volume [1, 7–11] and neuronal loss [12, 13], increase axonal sprouting [12] and revascularization [5, 6], as well as shifting the macrophage population towards a higher proportion of anti-inflammatory M2 macrophages relative to proinflammatory M1 macrophages [14]. This complex histological response suggests many possible targets for MSC influence on SCI recovery.

MSCs are multipotent cells capable of differentiating into cells of both neuronal and glial lineages [15–18], which can produce a wide array of trophic and anti-inflammatory factors [19–21]. In immunosuppressed rats, IV delivered MSCs can engraft into sites of spinal cord injury (SCI) [4] or brain ischemic injury [22]. However, in non-immunosuppressed animals, IV delivered MSCs were not detected at sites of spinal cord injury [1, 6], although they still promoted functional recovery. As in models of myocardial infarction [23], peritoneal inflammation [24], liver ischemia [25], and lethal radiation [26], MSCs, that are intravenously infused into non-immunosuppressed rats with SCI, are detected primarily in the lungs, where they are eliminated within 24–48 hours post-infusion [1]. The lack of detection of transplanted MSCs within the injured spinal cord implies that these stem cells promote recovery by releasing substances into the general circulation that are then able to mediate a therapeutic effect at the site of injury.

In several experimental injury models, including stroke [27], myocardial infarction [28, 29], liver toxicity [30, 31], kidney disease [32–34], and status epilepticus [35], the therapeutic effects of systemic MSC delivery could be replicated by transplantation of exosomes produced and secreted by MSCs (MSC^exos^; see [36] for a review). Furthermore, MSC^exos^ have been shown to modulate immune function *in vivo* [37] as well as to promote cortical neurite outgrowth [38] and endothelial cell proliferation, migration, and tube formation [28] *in vitro*, indicating that MSC^exos^ might be capable of mediating many of the histological changes observed after intravenous MSC infusion in SCI animals. We therefore postulated that exosomes might be the secreted factors responsible for the therapeutic effects of IV infused MSCs on SCI recovery.

Exosomes are nanovesicles (30–150 nm) with bilaminar membranes. They are among a family of extracellular vesicles (EV) released by all cells, of all species, and are present in all biologic fluids examined [39–41]. Exosomes are formed intracellularly by pinching off the walls of terminal endosomes and accumulate intracellularly in the multivesicular body (MVB) at the cell periphery before being released in bunches extracellularly upon exocytosis of the MVB. The biochemical signature of exosomes is distinct from other vesicles. Exosome membranes are notably enriched in sphingolipids and tetraspanin proteins such as CD63, and they contain a variety of RNAs; particularly mRNAs and microRNAs (miRNAs) [41–44], which can be transferred to targeted recipient cells, including neurons [27, 38, 45], to modulate gene expression in those cells [38, 46, 47]. Pilot studies in our lab [48, 49] and a recent report [2] suggest that IV delivery of MSC^exos^ could replicate some early therapeutic actions of IV delivered MSCs, but it was not clear whether MSC^exos^ trafficked to the injury site or which cell types might be targeted.

In the current study, we report that IV infused DiR labeled MSC^exos^, unlike IV infused MSCs, trafficked to contusive SCI sites, but not uninjured spinal cord. Furthermore, the IV infused DiR-labeled MSC^exos^, were specifically taken up by a subset of macrophages at the SCI site expressing the M2 marker CD206, but not by macrophages expressing the M1 marker iNOS. Importantly, the hotspots of DiR fluorescence within CD206^+^ M2-type macrophages strongly co-localized with staining for the characteristic exosome antigen CD63, supporting the identification of the fluorescence as clusters of exosomes, rather than nonspecific debris or dye accumulation. These findings argue that MSC derived exosomes may contribute to the therapeutic effects of IV administered MSCs on recovery from spinal cord injury by regulating the actions of macrophages within the lesion.

## Materials and methods

All experiments were carried out in accordance with NIH guidelines for the care and use of laboratory animals, and the VA Connecticut Healthcare System Institutional Animal Care and Use Committee approved all animal protocols. Animals were sacrificed under sodium pentobarbital anesthesia (80mg/kg i.p.) prior to tissue harvesting.

### Culturing and labeling of MSCs

MSCs were isolated from the bone marrow of young adult Sprague-Dawley rats (150-200g) as previously described in (Osaka et al. 2010), cultured in DMEM with 10% heat inactivated fetal calf serum (FCS), glutamate and penicillin/streptomycin and passaged when cells reached 70–80%. After the 4^th^ or 5^th^ passage (P4 or P5), MSC were washed 3 times in PBS and incubated with 10ug/ml DiR (1,1-dioctadecyl-3,3,3,3-tetramethylindotricarbocyanine iodide) (Molecular Probes) in DMEM for 10 minutes at 37°C in a culture incubator per manufacturer’s recommendations, or with RNA binding Syto RNASelect (Molecular Probes), or the fluorescent sphingolipid analogue BoDipy (Molecular Probes), for 30 minutes per manufacturer’s recommendations. Media containing the dye was aspirated and replaced with serum containing media for 3 hours to allow cells to recover before washing the adherent cultures in plain DMEM and transferring to serum free media for collection of exosome containing conditioned media.

Potential effects of DiR labeling on MSC viability were assessed by deplating 4 x 10^6^ P4 or P5 MSCs from 4 separate donors, incubating half of each cell suspension with 10 μg/ml DiR (Molecular Probes) for 10 minutes, and the other half in plain DMEM for the same length of time. Cells were washed once with DMEM and once with 10% fetal calf serum containing media, before resuspending in 2ml DMEM and counting cell samples with a hemocytometer. No reduction in the number of trypan blue excluding cells was detected in DiR treated samples from any of 4 separate tests (108 ± 4% of control (n = 4)). Cultures of MSCs were also examined before labeling and at the time of collection of conditioned media for exosome isolation. No reduction in densities of attached cells were noted in DiR labeled cultures compared to unlabeled cultures.

### Exosome isolation and characterization

**Fig 1:**
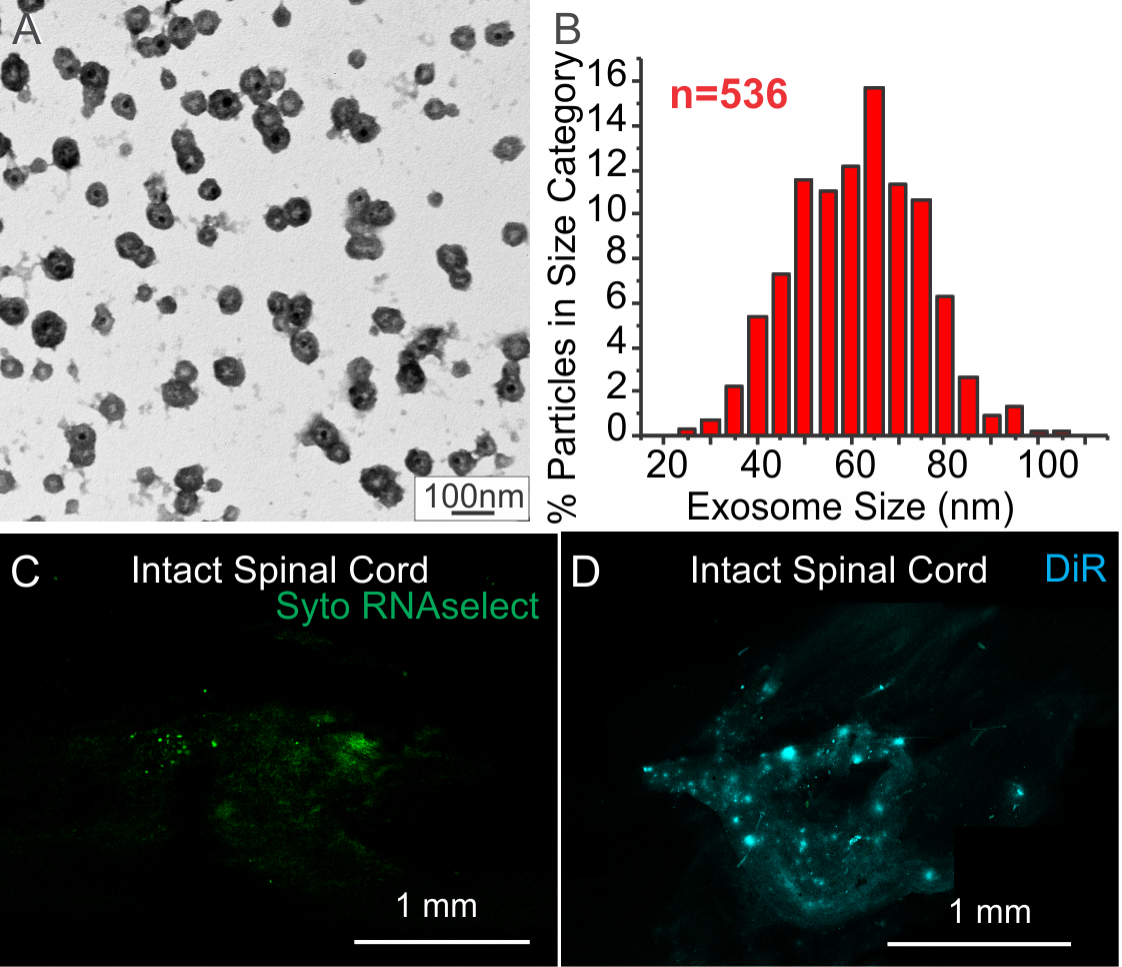
Exosome characterization. A: Electron micrograph of vesicles in exosome fraction from MSC conditioned media sample on formvar coated grid. B: Histogram of size distribution of 536 presumptive exosomes. Note the average size of the vesicles was 59.09 ± 0.58 nm, consistent with exosomes. C-D: Fluorescence micrographs of intact spinal cord directly injected with exosomes fractions from SytoRNAse (C) and DiR (D) labeled MSCs before perfusion. Note that the presence of detectable levels of 488nm (SytoRNAse) and 650nm (DiR) fluorescence indicates the presence of both RNAs and lipids in the exosome fractions. Scale bar in A indicates 100nm. Scale bars in C &D indicate 1 mm.

Exosomes were isolated from 2-day serum free conditioned media from MSC cultures using the differential centrifugation methods described by Théry et al. (2006), and used previously by our lab (Bryniarski et al 2013 and 2014). For one of the DiR labeled exosome transplant experiments, conditioned media was concentrated tenfold using Microsep 3K filter units prior to the final ultracentrifugation steps. Protein content of exosome fractions was assessed using a commercial Bradford protein assay kit (Thermoscientific) and Synergy HT plate reader (Biotek). Exosome numbers were assessed in two of the samples by nanoparticle tracking analysis (NTA) employing a NanoSight LM10 (Malvern Instruments, United Kingdom) with data extrapolated to other samples based the Bradford protein assay. Morphology of exosome vesicles was verified for some samples by electron microscopy (see Fig 1). For EM identification, exosome samples were fixed with 2% paraformaldehyde, adsorbed to Formvar coated copper EM grids, post-fixed with 1% gluteraldehyde, washed and stained with uranyl acetate and lead citrate before coating with a thin film of 2.5% polyvinyl alcohol and air drying (Tokuyasu et al 1989). Grids were examined and a random sample of exosomes were measured using a JEOL JEM-1011 electron microscope operating at 20KV and AMT-225 high resolution digital imaging system (Advanced Microcopy Techniques). Effectiveness of labeling was assessed by microinjection of 2–3 μl samples into spinal cords of non-contused animal 3 hours prior sacrifice, followed by local vascular perfusion to remove unattached material, and then examination of frozen sections.

### Rat spinal cord injury and DiR labeled MSC^exos^ IV infusion

Adult male Sprague-Dawley rats weighing 195-220g were deeply anesthetized with isoflurane (1–3%) inhalation anesthesia and subjected to contusion injury in the spinal cord using the Infinite Horizon (IH) impactor spinal cord injury device (Precision Systems & Instrumentation, Lexington, KY). Contusion injuries were induced by applying an impact of 2 Newton (equal to 200 kilodyne) at Th9 spinal cord level exposed by laminectomy as described previously [1].

One week post-contusion, DiR labeled MSC^exo**s**^ (100 μg protein estimated to be 2.5 ×10^9^ exosomes via NanoSight LM10in PBS) was infused (0.2 ml in PBS) into the saphenous veins of isoflurane anesthetized rats over the course of 1 minute. Control contused rats were infused with PBS only or PBS containing DiR.

Three batches of DiR-MSC^exos^ (derived from approximately 50 x10^6^ MSCs for each batch) were used for three separate infusion experiments. In each group, two animals were infused with DiR-MSC^exos^ and one animal was infused with an equal volume of PBS. One DiR-MSC^exos^ infused animal in each group was sacrificed at 3 hours and the other at 24 hours post infusion. The PBS infused control animals were sacrificed at 3 hours post- 24 post-infusion. A total of 6 contused animals were transplanted with DiR-MSC^exos^ and 3 contused animals were infused with PBS. Two additional contused animals were infused with PBS containing DiR at the same concentration used for labeling MSCs.

### Histological processing and staining of rat tissues and red blood cells

At 3 hours or 24 hours post DiR-MSC^exos^ or control PBS infusion, rats were sacrificed under sodium pentobarbital anesthesia, perfused with saline, followed by 4% paraformaldehyde in Sorreson’s phosphate buffer. Spinal cords, spleens, livers, lungs and testes were removed and processed for standard 15 μm frozen sectioning. One centimeter longitudinal sections from both intact and lesioned areas of the spinal cord, as well as the spleen, liver, lung, kidneys, and testis were either mounted directly in DAPi imputing media or stained with primary antibodies directed against one or more of the following: Neurofilament (chicken polycolonal 1:1000; Encor Biotech CPCA-NF H), GFAP (chicken polyclonal 1:1000; Encor CPCA-GFAP), RECA-1 (mouse monoclonal 1/50; AbD Serotec MCA970R), PDGFR-*B* (rabbit monoclonal 1:200; Cell Signaling Technologies 3169S), CD63 (1:100, SCI Systems Biosciences, ExoAB-CD63 A-1), OX-42 (1:100 BD Pharmingen 550299), CD206 (1:50 Santa Cruz Biotechnology Inc. sc-376108), iNOS (1:200 Abcam ab3523), CD4 (1:100, BD Biosciences 550298), CD8 (1:100, Bio-Rad MCA48R), and visualized with secondary goat anti-mouse, -rabbit, or -chicken IgG antibodies conjugated to Alexa Fluor 488, 546, 594, or 633 (Invitrogen, Eugene, OR; 1:1000). Immunostained sections or unstained sections were counterstained with DAPi mounting media (Vectashield, Vector Laboratories, Burlingame, CA) and photographed with a Nikon A1R multiphoton confocal microscope with NIS Elements software.

To assess fluorescence of fixed red blood cells, blood was collected form one animal in a heparinized tube at the time of sacrifice, centrifuged at 6,000RPM for 30 seconds with a desk top microfuge, resuspended and washed in PBS before fixing with 4% paraformaldehyde in Sorreson’s phosphate buffer for 15 minutes and washed twice in PBS. One drop of resuspended cells was added to a glass slide and allowed to partially dry and adhere to the surface before mounting with DAPi mounting media (Vectashield, Vector Laboratories, Burlingame, CA), cover slipping.

### Image processing and analysis

For imaging and analysis of stained and/or transplanted tissues, thresholds for each wavelength were adjusted to subtract autofluorescence levels at the same wavelength in unstained tissue of the same type which was washed and mounted with DAPI mounting media, or processed with secondary antibodies only and mounted with DAPI mounting media. Effects of contusive injury on auto fluorescence of spinal cord tissue in the lesioned area at the 488, 595, and 633 nm wavelengths were assessed by mounting unstained sections of intact and contused spinal cord tissue from 2 PBS infused animals, photographing three fields in the center of each section at 20X (403,061 μm^2^ area) and quantifying the average fluorescence for each wavelength using NIS Elements software which ranked intensity on a scale from 0 to 4092. Lesioned areas were identified by mottled appearance in the 488, 595 wavelengths and altered distribution of nuclei as seen in the DAPI wavelength. Average autofluorescence in both the 488 nm and 595 nm wavelengths increased by an average of 159 and 215 units, respectively, in the contused region of the spinal cord, compared to the intact portion of the same spinal cord. By comparison, average autofluorescence in the 633 nm DiR wavelength was increased by only 54.

To assess the relative distribution DiR hotspots within contused spinal cords, sections were stained with GFAP and, DiR hotspots were counted within 10 nonoverlapping fields within the lesioned area (as defined by the reactive astrocyte border), and 10 nonoverlapping fields at least 100 μm outside of the reactive astrocyte boundary within the same section as well as 10 nonoverlapping fields in sections of the spinal cord taken more than 1 cm rostral to the lesion. Three different sections were analyzed from two different contused and DiR-MSC^exos^ transplanted animals and one DiR only infused animal. Sampled areas outside of the contusive lesion and in nonlesioned spinal cord included both grey matter and white matter areas.

To evaluate relative sizes of DiR hotspots within contused spinal cord tissue at 3 hours and 24 hours post-infusion, compact areas of DiR fluorescence were measured at their longest diameter in 20X images of spinal cords harvested at 3 and 24 hours post-infusion using NIS Elements software. DiR hotspots were measured for all concentrated areas of DiR fluorescence, regardless of intensity or proximity to other hot spots, using the DiR channel only form the original image. A total of 260 and 524 hot spots respectively were measured for a total of 16 separate images of varying sizes from 3 and 24 hour post-contusion spinal cords

### Spleen weights

To assess the gross effects of spinal cord injury on mobilization of circulating monocytes from the spleen, spleens from 1 week post SCI rats and age and sex matched controls were removed, washed with PBS, blotted to remove excess fluid and weighed.

Spleen weights for control and spinal cord contused rats were compared using and one-way Analysis of variance (ANOVA) with a post-hoc test for multiple comparisons.

### Statistical analysis

All statistical comparisons were performed using Origin software (version 8.1; OriginLab Corporation, Northampton, MA), and one-way Analysis of variance (ANOVA) with a post-hoc test for multiple comparisons. Comparisons which are reported as statistically significant were significant at p<0.05 or lower.

## Results

### Confirmation of exosome isolation and labeling

**Fig 2:**
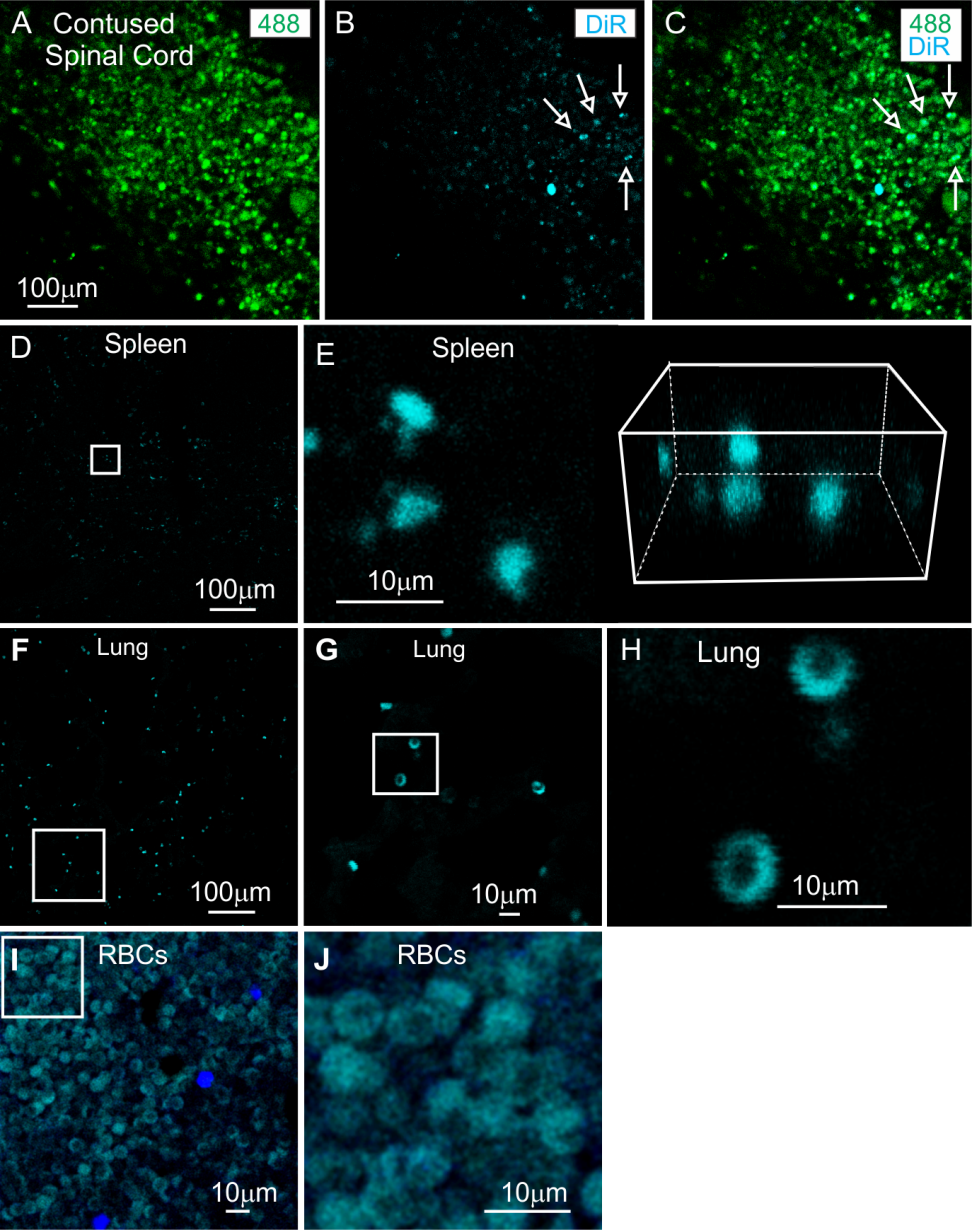
Exosome detection in situ. A-C: Two photon confocal micrographs of contused spinal cord 3 hours after IV infusion of DiR labeled exosomes showing the colocalization of regions of high endogenous 488 autofluorescence with 650nm DiR hotspots. D-H: Endogenous DiR wavelength in the spleen (D-E) and lungs (F-H) of contused animals with no exosome infusion. High magnification shows sizes and shapes of DiR hotspots in match those of red blood cells. I-J: Endogenous DiR wavelength fluorescence of paraformaldehyde fixed rat red blood cells isolated from peripheral blood. Scale bar in A indicates 100 μm and applies to A-C. Scale bars in D, F indicate 100 μm and applies to A-C. Scale bars in D, F indicate 100 micrographs of contused spinal cord 3 hours DiR hotspots.

Electron microscopy confirmed the presence of high concentrations of 30–100 nm diameter vesicles, consistent with the morphology of exosomes within the isolated 100,000g pelleted exosome fraction from bone marrow mesenchymal stem cell (MSC) conditioned media (Fig 1A and 1B). Exposing cultured MSCs to fluorescent markers for RNA (Syto RNASelect, Fig 1C), exosome specific sphingolipids (BODIPY, not shown), or nonspecific membrane lipid (DiR; Fig 1D) resulted in fluorescent labeling of exosomal fractions from conditioned media, which could be detected after direct injection into intact spinal cord tissue. Examination of droplets of DiR, Syto RNASelect, or BODIPY labeled exosome solutions with the confocal microscope showed essentially uniform fields of low level fluorescence with no evident structure, consistent with particles too small to detect at the light microscope level (not shown). Although the effectiveness of the three methods of staining indicated that the composition of the exosomal fraction was consistent with that of exosomes, fluorescence intensity was consistently higher using DiR labeling protocols (Fig 2A and 2B). Furthermore, when detection thresholds for dye visualization were adjusted to subtract the level of fluorescence observed within contused spinal cord lesions, rather than the background level for the intact spinal cord, more than half of the areas of Syto RNASelect and BODIPY fluorescence were lost, while only the area of DiR fluorescence was minimally reduced, indicating that concentrations of Syto RNASelect, or BODIPY labeled exosomes would be more difficult to identify in the contused spinal cord than DiR labeled exosomes.

### In situ detection of DiR-labeled exosomes

Spinal cord injury sites exhibited relatively high levels of autofluorescence in the 488 nm green (Fig 2A) and 562 nm red (not shown) wavelengths, consistent with the presence of large numbers of autofluorescing macrophages in these regions [50, 51]. Examination of spinal cords of DiR-MSC^exos^ IV infused SCI rats revealed numerous hotpots of DiR wavelength fluorescence within lesion zones at 3 hours (Fig 2B) and 24 hours (see below) post-IV infusion which colocalized with these areas of high autofluorescence (Fig 2C). DiR hotspots were extremely rare or absent in intact regions of the spinal cord in DiR-MSC^exos^ infused animals, either rostral or caudal to the impact sites (not shown). Counts from ten sample fields of DiR hotspots within and outside of the contused areas found an average of only 0.66 ± 0.33 DiR hotspots outside of the lesion zones as compared to 44.33 ± 0 14.11 DiR hotspots within the lesion area (p<0.05 ANOVA). Counts were obtained from three 1 cm longitudinal sections from two separate contused rats.

No DiR wavelength hotspots were detected in sections taken from spinal cord tissue greater than 1 cm rostral to the lesion site 0±0 (n = 3 sections) or in the lesioned area of an animal infused with DiR only without exosomes 0±0 (n = 3 sections). Unlike 488 nm (green) and 562 nm (red) wavelengths, fluorescence in the far red 640 nm wavelength of DiR was rarely observed in contused regions of the spinal cords or other tissues or organs from control PBS infused animals. When DiR wavelength hotspots were detected in control IV infused tissue, typically spleen (Fig 2D and 2E) or lung (Fig 2F–2H) sections, DiR wavelength fluorescence was associated almost exclusively with small, anucleated, discoidal structures, consistent with fixed rat red blood cells (RBCs), that were found to emit autofluorescence in this wavelength (Fig 2I and 2J).

### IV infused DiR-MSC^exos^ localize to macrophages in the contused spinal cord

**Fig 3:**
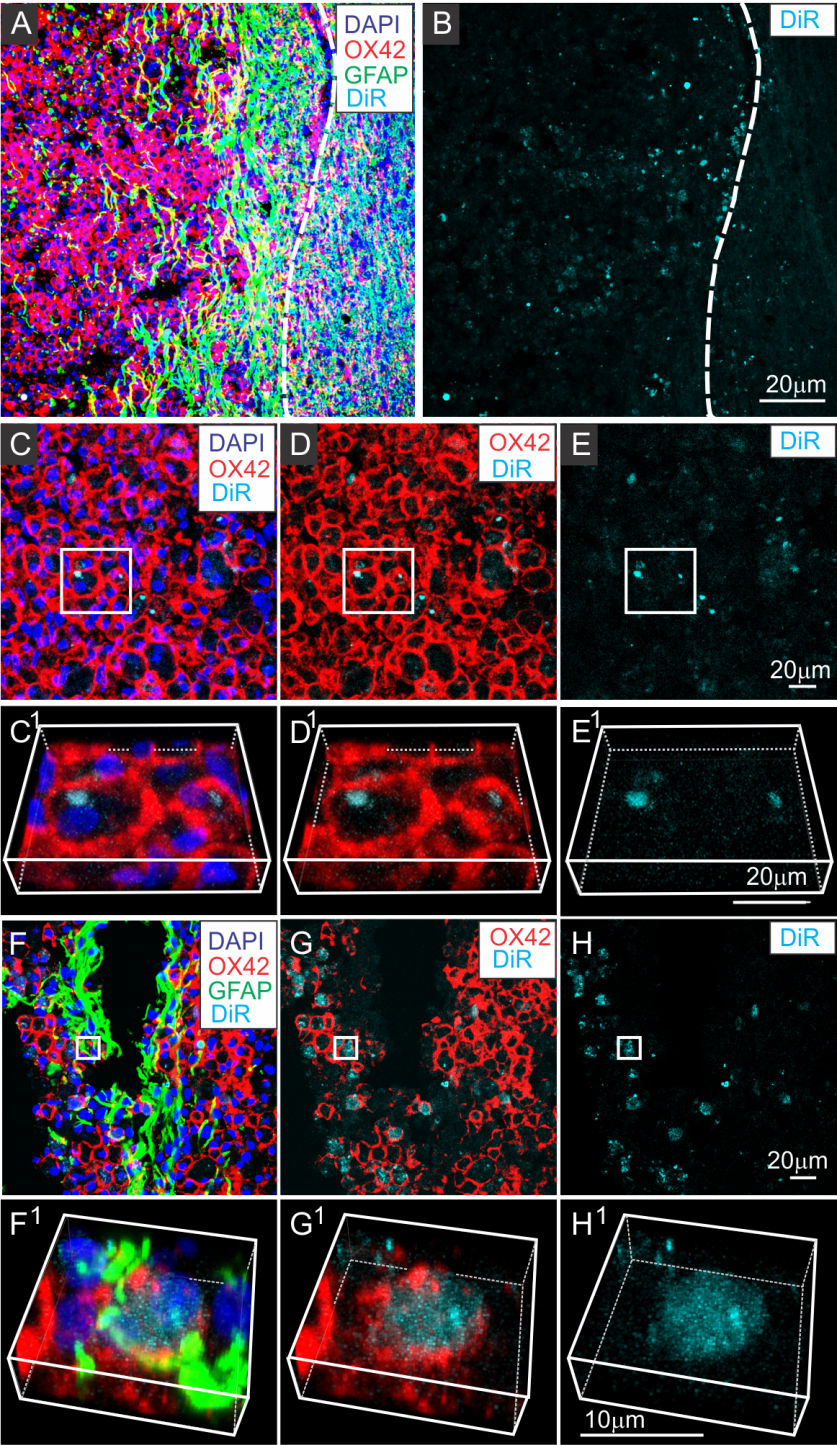
IV infused DiR labeled MSC^exos^ co-localize with OX42^+^ macrophages/microglia in the contused spinal cord. A-B: Low magnification confocal images of a representative region of a contused spinal cord 3 hours after DiR MSC^exos^ infusion stained for GFAP (green) and OX42 (red) and counterstained with DAPI (blue) (A) or DiR fluorescence only (cyan) (B). Large numbers of DiR hotspots can be seen near the margins of the contusion site correlating with high levels of GFAP^+^ reactive astrocytes (green) and OX42^+^ macrophages (red). C-E: Higher magnification image of the lesion shown in A showing the relationship between DiR hotspots and OX42 staining. C^1^-E^1^: Enlarged and rotated boxed regions of the lesion shown in C-E showing that the location of DiR hotspots within OX42^+^ cells. F-H: Representative confocal images of contused spinal cord 24 hours after DiR MSC^exos^ (cyan) infusion shown stained for GFAP (green) and OX42 (red) and counterstained with DAPI (blue), (F), OX42 and DiR (G) or DiR fluorescence only (cyan) (H). F^1^-H^1^: Enlarged and rotated boxed regions of the lesion shown in F-H. All scale bars indicate 20 μm. Dashed line in A and B indicates the approximate border of the lesion, as defined by the reactive astrocyte boundary, with the lesion to the left and largely intact neural tissue to the right. DiR hot spots appear to be clustered more densely near the borders of the lesioned area, in regions containing both OX42^+^ macrophages (red) and strongly GFAP^+^ reactive astrocytes (green).

Unlike control PBS or DiR solution infused animals, hotspots of DiR fluorescence could be readily identified within SCI sites of DiR-MSC^exos^ IV infused rats. Large numbers of DiR fluorescence hotspots were detected within contused lesions at both 3 hours (Fig 3A–3E) and 24 hours (Fig 3F–3H) after IV infusion of DiR-MSC^exo**s**^ into 1-week post-SCI rats. The distribution of DiR labeling was not random. DiR hotspots were concentrated predominantly near the margins of the lesions, as defined by the boundary between the OX42^+^ macrophage-rich phagocytic zone and the GFAP^+^ astrocyte-rich glial scar (Fig 3A and 3B; see dashed line). These DiR hotspots were consistently associated with OX42^+^ macrophages/microglia (Fig 3C–3H). Three-dimensional rotation of images confirmed that essentially all DiR-MSC^exos^ hotspots were localized within macrophages (Fig 3C^1^–3E^1^, 3F^1^–3H^1^), and not associated with anucleated RBCs or other cell types in the immediate area.

The pattern of DiR fluorescence distribution within spinal cord lesions suggested an increasing accumulation of DiR-MSC^exos^ within individual cells over time. Although only a fraction of OX42^+^ macrophages/microglia within the lesions contained DiR hotspots, the proportion of the DiR containing macrophages did not noticeably increase or decrease between 3 hours (Fig 3C–3E) and 24 hours (Fig 3F–3H) post-infusion of DiR-MSC^exos^. However, the sizes of DiR hotspots appeared noticeably larger at 24 hrs. post-infusion (Fig 3F–3H) than at 3 hrs. post-infusion (Fig 3C–3E). In many cases, DiR fluorescence completely filled the cytoplasm of OX42^+^ cells examined at 24 hrs. after DiR-MSC^exos^ infusion (Fig 3F–3H). Average diameters of DiR hot spots in contused spinal cords of animals sacrifice 24 hours after DiR-MSC^exos^ infusion were significantly larger than DiR hot spot diameters in animals sacrificed at 3 hours post-infusion (6.70 ± 0.15 μm n = 524 hotspots compared with 5.47 ± 0.18 μm n = 260 hotspots; p<0.05 ANOVA.), with many cells at the 24 hr time point containing more than one hot spot.

**Fig 4:**
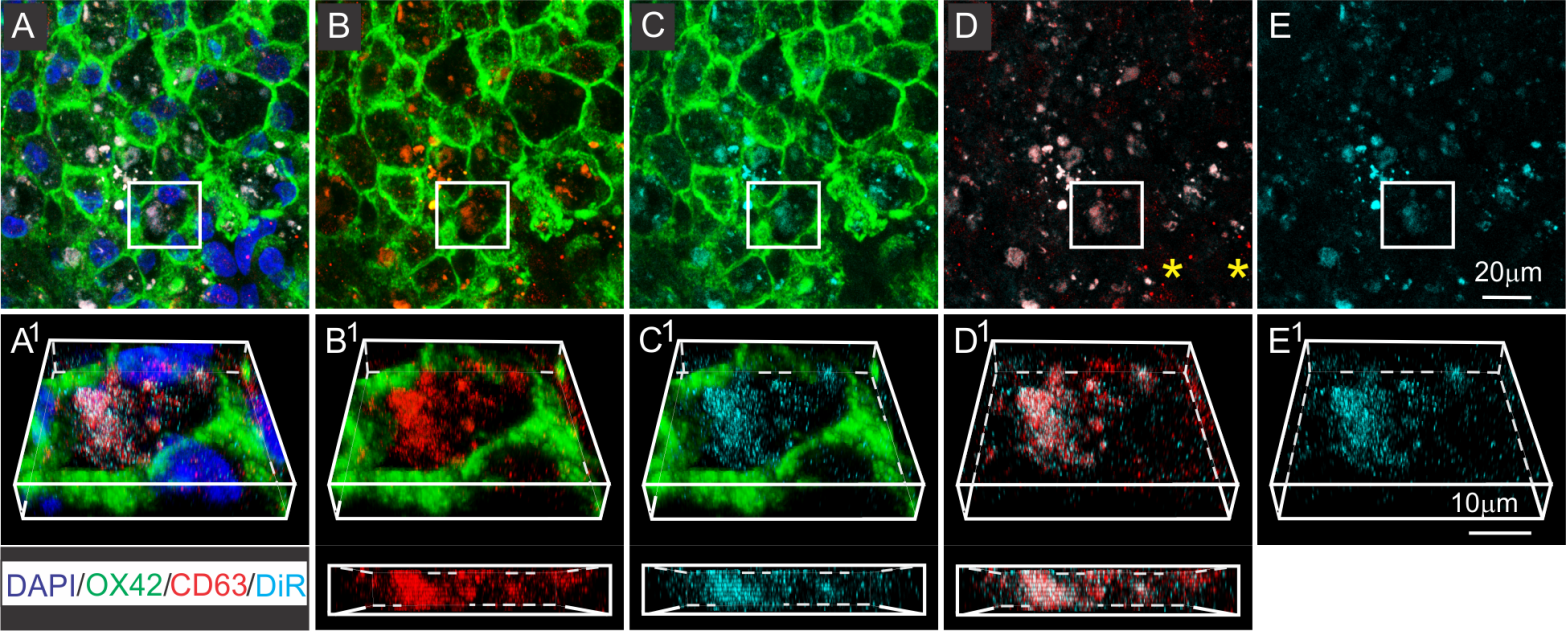
DiR hotspots in DiR-MSC^exos^ infused animals co-localize with CD63 exosome marker. A-E: Representative region of a contused spinal cord 24 hours after DiR MSC^exos^ (cyan) infusion stained with antibodies directed against OX42 (green) and the tetraspanin CD63 (red), and counterstained with DAPI (blue) (A) or DiR fluorescence only (cyan). Images show all wavelengths (A), OX42 & CD63 (B), OX42 & DiR (C), CD63 & DiR (D), DiR only (E). A^1^-E^1^: enlarged and rotated sections of boxed regions shown in A-E. Images below B^1^, C^1^, and D^1^ respectively show full 90 degree rotations of boxed regions showing CD63, DiR, and CD63 & DiR fluorescence. Note the strong co-localization between DiR fluorescence and CD63 staining. Scale bar in E = 20 μm E^1^ = 10 μm.

Co-staining with antibodies directed against OX42 and the exosome enriched tetraspanin CD63 supported the identification of DiR hotspots within macrophages as exosomes. CD63 staining within OX42^+^ macrophage rich areas strongly correlated with locations of DiR hot spots (Fig 4). Although some CD63 stained patches showed no DiR wavelength fluorescence (Fig 4D), all DiR fluorescence hotspots corresponded to patches of CD63 staining (Fig 4D and 4E), implying that all DiR hotspots contained exosomes, but that not all exosomes came from the infused DiR-MSC^exos^. The close association between DiR fluorescence and CD63 staining was maintained when images were rotated (Fig 4A^1^–4E^1^), more definitively demonstrating that the anti-CD63 antibody stained the same element which expressed the DiR wavelength fluorescence.

### DiR-MSC^exos^ localize specifically to Type 2 macrophages (M2) and not Type 1 macrophages (M1)

**Fig 5:**
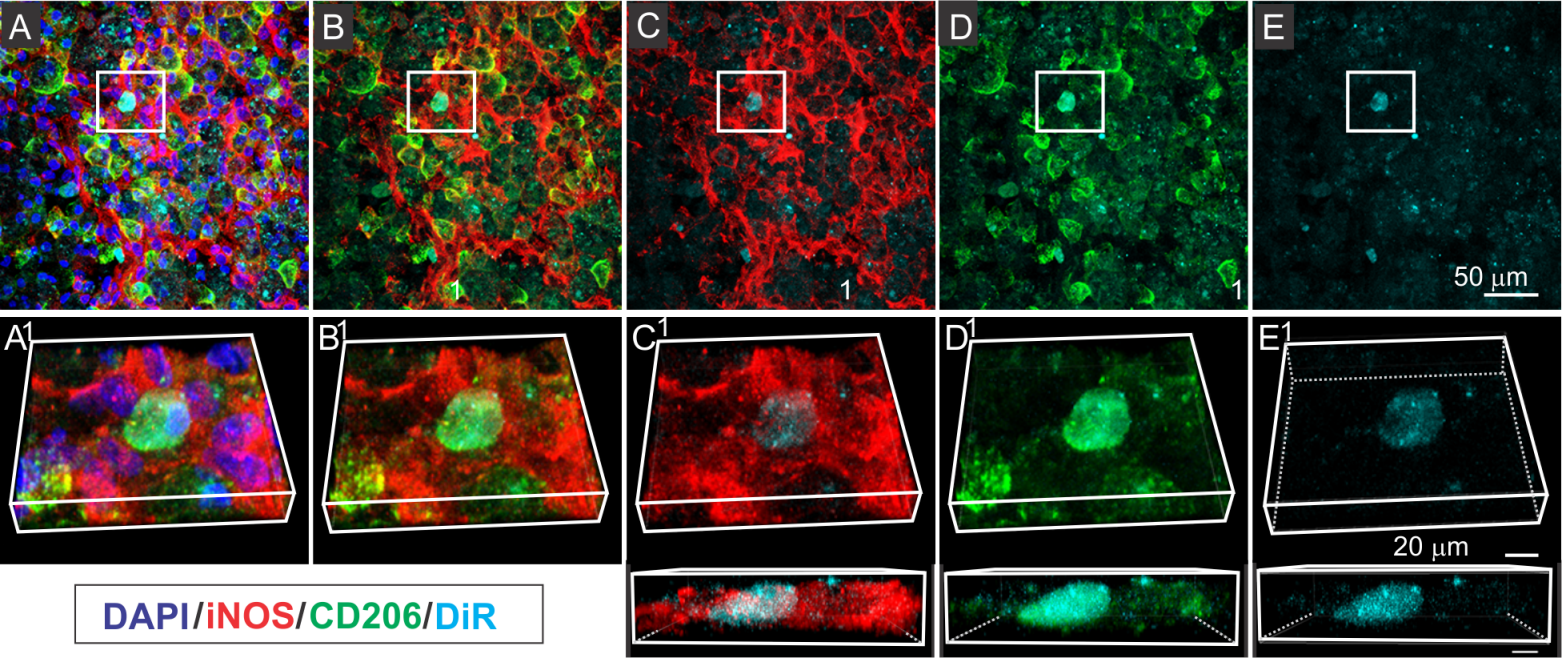
IV infused DiR labeled MSC^exos^ localize with CD206^+^ Type M2 macrophages in the contused spinal cord. A-E Confocal micrographs of a representative region of a frozen sectioned contused spinal cord harvested 24 hours after IV infusion of DiR labeled exosomes (cyan), immunostained with antibodies directed against Type M1 macrophage marker iNOS (red) and Type M2 macrophage marker CD206 (green) and counterstained with DAPI (blue). Images from left to right show the same area showing florescence channels for (A) iNOS, CD206, DAPI, & DiR, (B) iNOS, CD206, & DiR (C) iNOS, & DiR, (D) CD206, & DiR, and (E) DiR only. Images in a-e show enlarged images of boxed area above rotated and illustrated in 3D. A smaller portion of the regions shown in c-e is further enlarged and rotated below. Note the strong co-localization of DiR florescence with CD206, but not iNOS staining, suggesting that DiR labeled exosomes are taken up by Type 2 macrophages. Scale bars in E and e & d indicate 50 μm and 20 μm and apply to A-E and a-e respectively.

Spinal cord sections stained with antibodies directed against different macrophage subtypes indicated that DiR fluorescence was associated specifically with the M2, but not the M1 macrophages (Fig 5). Contused regions of spinal cord contained high numbers of both iNOS^+^ M1 macrophages and CD206^+^ M2 macrophages (Fig 5A, 5B, 5A^1^ and 5B^1^). However, DiR hotspots from the DiR-MSC^exo**s**^ infused animals were consistently observed within CD206^+^ M2 macrophages (Fig 5D and 5D^1^), but not within iNOS^+^ M1 macrophages (Fig 5C and 5C^1^). This specificity of cellular localization was particularly apparent in rotated images of individual “DiR hot spots”, which in some cases showed an almost perfect overlap between CD206 staining and DiR fluorescence (top and bottom of Fig 5C^1^–5E^1^).

Although only a fraction of CD206^+^ M2 macrophages exhibited DiR fluorescence, virtually all DiR hotspots detected in iNOS/CD206 double stained section were localized to CD206 positive M2 cells (Fig 5C). DiR fluorescence was extremely rare within iNOS^+^ M1 type macrophages. Of a total of 215 DiR hot spot containing cells identified in a retrospective analysis of 23 micrographs of spinal cord lesions, 213 DiR hot spot containing cells (99%) were CD206^+^ and only 2 DiR hot spot containing cells (less than 1%) were iNOS^+^.

### Lack of detection of DiR-MSC^exos^ in other cell types

**Fig 6:**
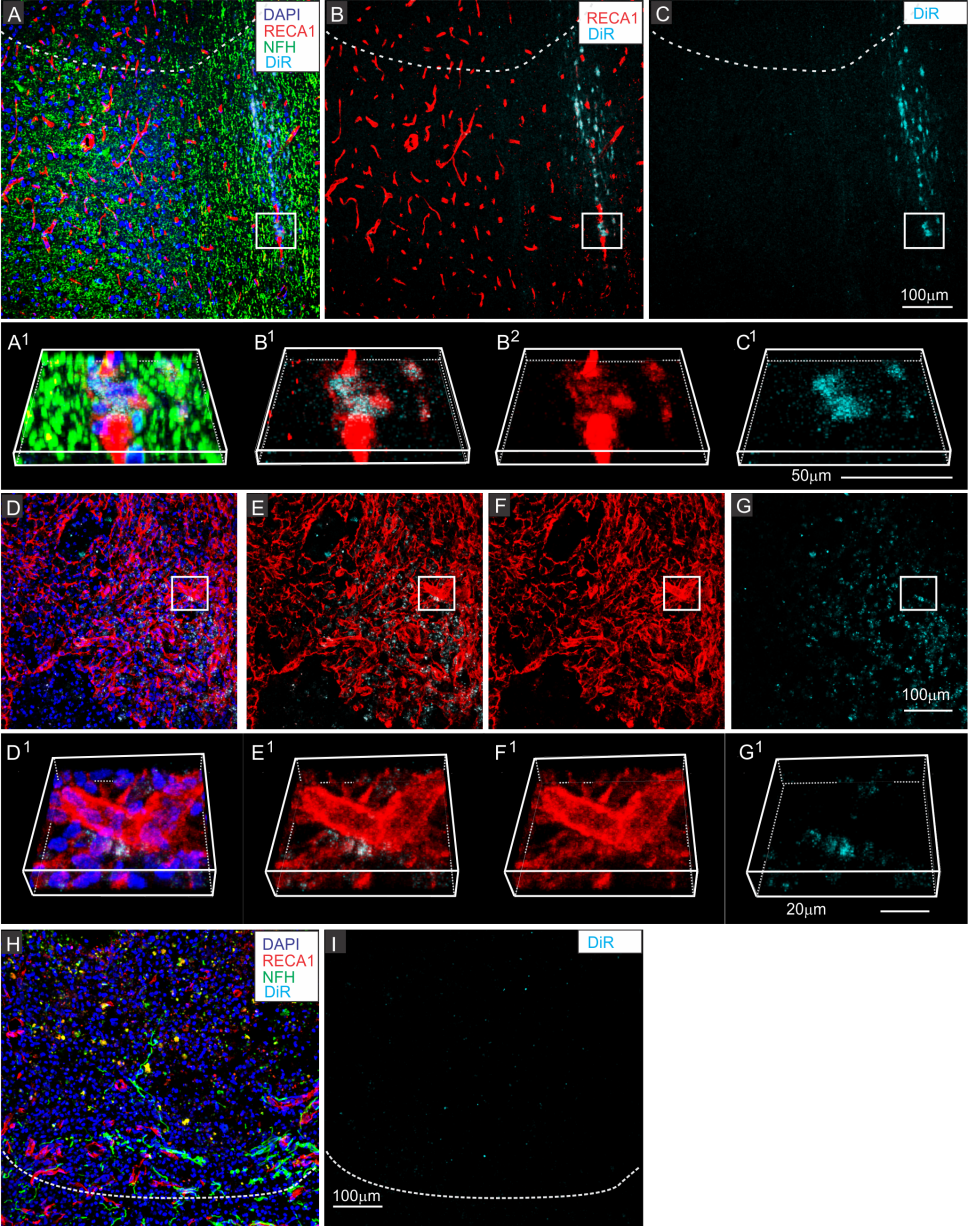
DiR hotspots not detected in other cell types at the lesion site. Selected regions contused spinal cords 24 hours after infusion of DiR MSC^exos^ (A-G) or PBS with free DiR (G-H), showing close proximity between DiR hotspots and some endothelial cells (A-C) and pericytes (D-G) within the central scar tissue at the contusion site and paucity of DiR fluorescence signal in DiR only infused animal. A-C: Region near the border of the lesion with antibody staining directed against neurofilament (NDH green), the endothelial cell marker RECA-1 (red) (A), RECA-1 (red) and DiR wavelengths (B), or DiR fluorescence only (cyan) (C). A^1^-C^1,^ B^2^: enlarged and rotated boxed region shown in A-C. Note that DiR hot spots are very close to the endothelial cells, but not within them. D-G: Selected region in the center of the contusion site containing high concentrations of pericytes Area is shown with antibody staining directed against neurofilament (green), PDGFR-*B* (red), DiR wavelength fluorescence (cyan) and DAPI counterstaining (blue) (D), PDGFR-*B* (red) and DiR wavelength fluorescence (cyan) (E), PDGFR-*B* (red) (F), or DiR wavelength fluorescence only (cyan) (G) D^1^-G^1^: enlarged and rotated boxed region shown in D-G. Note that DiR hotspots are located close to pericytes but are not within the pericytes. H-I: Representative region of the lesion in a contused rat infused with DiR only, without exosomes and stained with antibodies directed against RECA-1 (red), and neurofilament (green) and counterstained with DAPI (G) and the same regions showing DiR wavelength fluorescence only. Dashed lines in A-C and H, I indicate the approximate lesion boundary with the lesioned area above and intact spinal cord tissue below. Scale bars in A, G & I = 100μm. C^1^ 50 μm, E^1^ = 20 μm.

Immunostaining of lesioned spinal cord tissue with cell type specific antibodies directed at neurons (NFH), astrocytes (GFAP), endothelial cells (RECA-1) and pericytes (PDGFR-*B*) failed to show convincing evidence of exosome uptake by cells other than macrophages. Although DiR hot spots were sometimes detected in close proximity to degenerating blood vessels (Fig 6A–6C) or pericyte scar accumulations [52] in the centers of lesions (Fig 6D–6G), when individual cells were rotated to more precisely identify the locations of the hot spots, hot spots were consistently found to be outside of the RECA positive endothelial cells (Fig 6A^1^–6C^1^) or PDGFR-*B* positive pericytes (Fig 6D^1^–6G^1^).

Spinal cords of animals infused with a control solution of PBS and DiR (no exosomes) showed only small random specks of DiR wavelength fluorescence within the lesioned area (Fig 6H–6I) indicating that the dye itself did not specifically traffic to the injury site and that the apparent macrophage specific localization of the DiR host spots was not due to a specific affinity of macrophages for the dye.

### DiR fluorescence in other organs

**Fig 7:**
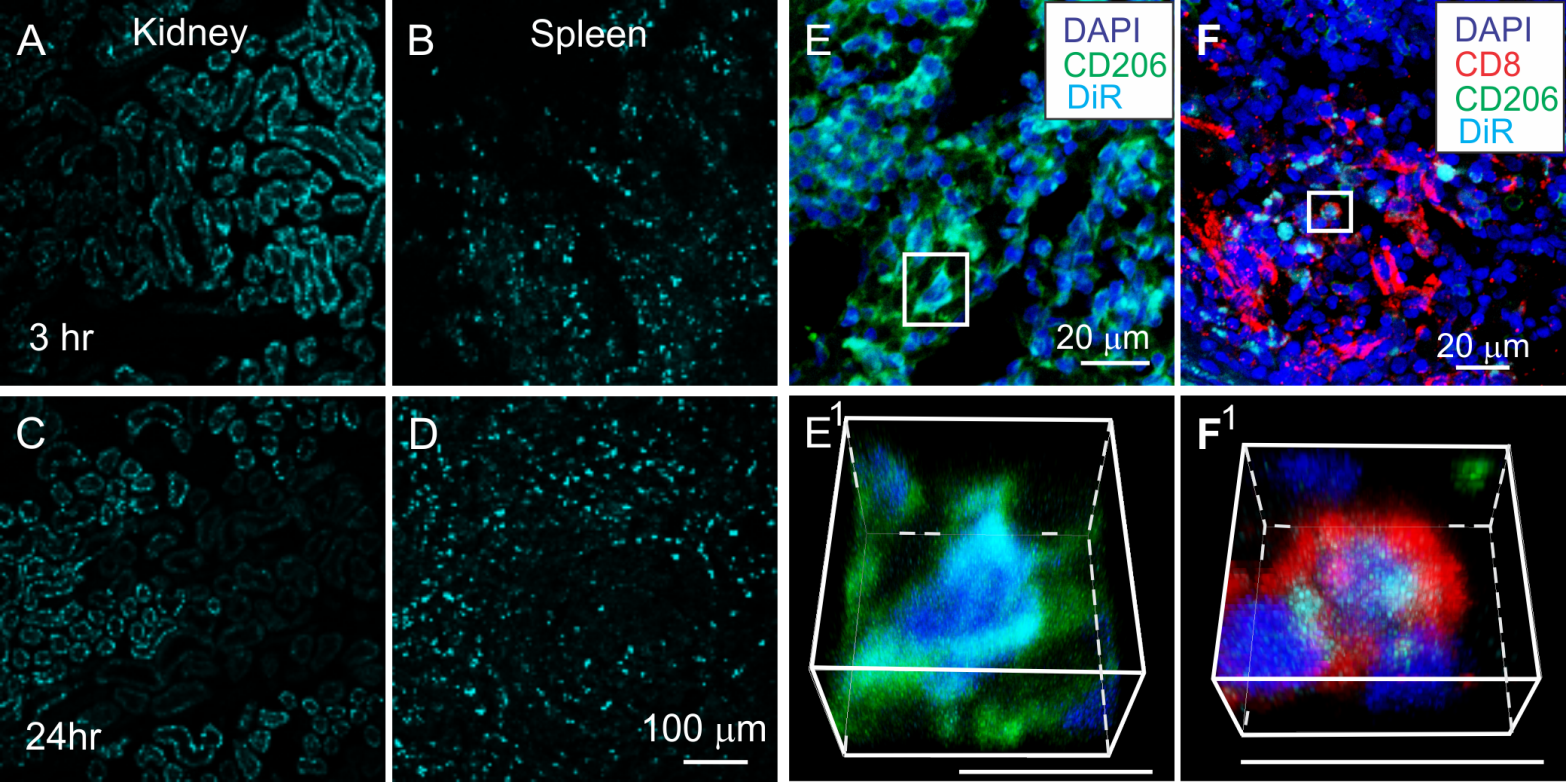
DiR hotspots transiently localize to kidney and more persistently to spleen. A-D: DiR fluorescence (cyan) in kidney (A, C) and spleen (B, D) 3hours (A, B) and 24 hours (C, D) after infusion with DiR-MSC^exos^. Note the decrease in DiR fluorescence in the kidneys between 3 and 24 hours after DiR-MSC^exos^ infusion, but increase in the spleen during the same time period. E-F: Spleens at 24 hours after DiR-MSC^exos^ infusion in SCI rats immunostained with antibodies directed against CD206 (green) and CD8 (red) DiR fluorescence hotspots. E^1^-F^1^: Enlarged rotated images of boxed areas in E and F. Note that DiR hotspots localize to both CD206^+^ and CD4^+^ cells in the spleen. Scale bars in E 100 μm and applies to A-D. Scale bars in E&F indicate 20 μm.

Diffuse DiR fluorescence was also observed in the livers (not shown) and kidneys (Fig 7A and 7C) of animals at 3 hours post-infusion (Fig 7A), which was significantly reduced at 24 hrs. post-infusion (Fig 7C). This reduction in DiR fluorescence in the kidneys and livers between the 3 and 24 hours post-infusion time points differed significantly from the increased fluorescence observed in the contused spinal cord, and implied a possible clearance of exosomes and/or DiR through the liver and kidneys rather than a specific uptake of DiR-MSC^exos^ by cells in these organs.

Large numbers of DiR hotspots were also detected within the spleen at both 3 (Fig 6B) and 24 hours (Fig 7D) after DiR-MSC^exos^ infusion. Similar to the contused spinal cord, sizes of DiR hotspots in the spleen tended to be larger at 24 hours after DiR-MSC^exos^ infusion (Fig 7D), than at 3 hours (Fig 7B) post infusion but were highly variable in size and density in the spleen. Furthermore, the spleen had a higher background level of auto fluorescence in this wavelength, likely due to the role of the spleen in degrading red blood cells which do fluoresce in the DiR wavelength. As in the spinal cord, some of the DiR hotspots in the spleen were localized to CD206^+^ cells (Fig 7E and 7E^1^). However, unlike the spinal cord, DiR hot spots in the spleen were not consistently associated with a single identifiable cell type. At least some DiR hotspot in the spleen were associated with CD8^+^ cells (Fig 7F and 7F^1^), and many DiR hotspot containing cells co-stained with more than one phenotypic marker (not shown). Analysis of DiR localization in the spleen was further complicated by higher background levels of patchy DiR wavelength autofluorescence in the spleen, possibly associated with the role of the spleen in breaking down red blood cells, which were the major source of DiR wavelength autofluorescence in other organs. Thus, the full range of the cellular targets of DiR-MSC^exos^ in the spleen remains uncertain. Detection of DiR fluorescence in lungs and testes was rare in DiR-MSC^exos^ infused animals and was associated almost exclusively with anucleated structures, consistent with autofluorescence of red blood cells as in PBS infused animals (Fig 2D–2H).

### Reduction in spleen weight after SCI

The superficial appearances of spleens of 1-week post-SCI rats suggested that they were smaller and paler with a more deflated appearance than spleens of control animals of the same age and weight or spleens of contused animals at 10 weeks post-SCI. Measurements of wet weight of spleens confirmed this visual impression, revealing an 18% reduction in spleen weight in 1 week post SCI rats, compared to those of age and weight matched control animals or SCI animals sacrificed at 10–12 weeks post SCI. Spleens of 1 week post-SCI animals averaged 685 ± 22mg (n = 13), compared to 839 ± 23 mg (n = 8) for intact animals and 874 ± 30 mg (n = 14) for 10–12 weeks post-SCI animals (p<0.05, ANOVA).

## Discussion

The results of this study show that IV infused MSC^exos^, unlike IV infused MSCs, traffic to the spinal cord injury site in non-immunosuppressed rats. Although the number of contused animals transplanted with MSC^exos^ was relatively small (6 animals), due to the need for large numbers of MSCs to produce a concentration of submicroscopic DiR-labeled MSC^exos^ that could be reliably detected after cellular uptake *in vivo*, the localization or the DiR labeled MSC^exos^ was extremely consistent. Specifically, DiR labeled MSC^exos^ were taken up by M2 macrophages within the lesion site. These findings suggest that intravenously delivered exosomes derived from MSCs, target the spinal cord injury site and may contribute to the therapeutic effects of IV infused MSCs in spinal cord injury.

The morphology and staining of the MSC^exos^ used in this study were consistent with identification of the infused MSC^exos^ samples and fluorescent “hotspots” within spinal cords as MSC derived exosomes. Sizes of individual vesicles in exosomal fractions observed with electron microscopy were consistent with size profiles of exosomes. Staining of exosomal fractions by prelabeling MSCs with Syto RNASelect, BODIPY, or DiR, and colocalization of DiR hotspots in tissue sections with CD63 staining also indicated that the presumptive exosomes contained the three characteristic components of exosomes: RNAs, sphingolipids, and tetraspanins, as well as nonspecific membrane lipids. The close correlation between DiR fluorescence hotspots and CD63 (tetraspanin) staining within macrophages is particularly significant as it argues that the fluorescence represented intact exosomes rather than residual dye from phagocytized particles. However, CD63 is also present on other internal vesicles [53, 54] and the presence of CD63 staining alone cannot definitively identify the presence of exosomes.

Tracking of DiR labeled MSC^exos^ to specific cells in the spinal cord demonstrates the feasibility of DiR labeling as a means of assessing the *in vivo* targets of systemically delivered exosomes, even in tissue with high endogenous autofluorescence, such as the macrophage rich areas associated with tissue injury. Endogenous fluorescence in the 640 nm wavelength for DiR was only observed in red blood cells, which could be readily identified by their distinct morphology and lack of nuclear staining. While others have demonstrated the feasibility of *in vivo* tracking of exosomes labeled with other membrane dyes such as PKH26 [35, 55, 56] and PKH67 (Fitzner et al., 2010), in our hands PKH26 and PKH67 were more cytotoxic to MSCs than DiR, potentially requiring dye labeling after exosome isolation, which has a higher risk of carryover of unbound dye in transplanted samples. No loss of viability was detected after DiR labeling of four batches of dissociated MSCs. However, a variable degree of cell loss was observed after PKH26 or PKH67 labeling in preliminary tests.

The three-dimensional rotation of confocal images in this study allowed unambiguous localization of DiR labeled exosomes within M2 macrophages of contused spinal cords. Unlike earlier studies using two-dimensional imaging of labeled exosomes *in vivo* [35, 55–57]), the 3-dimensional confocal images in this study left no doubt that the exosomes were internalized by the cells. Pericytes not associated with blood vessels have been demonstrated to represent a large number of cells in the glial scar after spinal cord injury [52]. In contrast to the macrophages, DiR hotspots were found in close proximity to some RECA-1+ endothelial cells and some PDGFR-*B*+ pericytes located within the central scar region of the lesion, but did not appear to be localized within these cells.

Although DiR-MSC^exos^ were specifically localized to macrophages within the contused spinal cord, it is not clear whether the DiR-MSC^exos^ were taken up by macrophages at the lesion site or whether macrophages migrated to lesion sites after taking up the exosomes. A circulating population of monocytes continuously cycles in and out of the splenic reservoir and migrates through blood vessels to sites of local inflammation [58, 59]. Furthermore, the reduced weights of spleens of contused rats suggests that there is an increased mobilization of monocytes after spinal cord injury, similar to that observed after major cerebral artery occlusion in the rat [60], although the spleen weight reductions we observed after SCI were smaller than those reported in the stroke model. Since only a fraction of the CD206^+^ macrophages in the spinal cord exhibited DiR fluorescence, and many DiR hotspots were detected within the cells in the spleen, it is possible that DiR-MSC^exos^ containing cells in both organs were derived from circulating monocytes, which took up exosomes in the blood stream and subsequently migrated into these organs. Alternatively, the cells may have taken up exosomes in the spleen and then migrated to the spinal cord.

The absence of DiR fluorescence in microglia outside of the lesion zone may indicate that exosome containing macrophages are not derived from resident microglia or that microglia/macrophages only take up exosomes after they become activated and/or migrate to the injury site. Although the blood spinal cord barrier (BSCB) becomes severely compromised immediately after SCI [1], the BSCB outside of the lesion area does not become significantly compromised until several weeks after contusive injury [1]. It is therefore possible that the intact BSCB prevented the administered exosomes from reaching microglia in the noninjured spinal cord areas, while these exosomes could more readily reach the parenchymal of injured spinal cord. Alternatively, since only a subpopulation of M2 macrophages in the lesion took up the exosomes, it is possible that only macrophages in a particular activation state can take up these exosomes. However, the apparent general increase in DiR cluster sizes for all hotspot containing cells at 24 hours post-infusion, compared to 3 hours post-infusion, suggests that macrophages continue to take up DiR-MSC^exos^ within the lesion site, rather than taking up exosomes only in the blood stream and retaining the same level of DiR-MSC^exos^ accumulation after migrating into the contused spinal cord.

The effect of MSC^exos^ uptake on macrophage function is uncertain. Exosomes produced by metastatic cancer cells have been shown to alter cell adhesion and promote neovascularization by endothelial cells [61, 62], and exosomes produced by stimulated macrophages and dendritic cells have been found to induce granulocyte migration [63]. But, in most cases, the effects of exosomes on targeted cells have not been determined. Since DiR-MSC^exos^ were only detected in CD206^+^ M2 macrophages, and not iNOS^+^ M1 macrophages, at both 3 hour and 24 hours post-infusion, it appears unlikely that uptake of MSC^exos^ results in conversion of macrophages from a pro-inflammatory M1 to anti-inflammatory M2 activation state. Furthermore, preliminary counts did not show an increase in the ratio of M2/M1 macrophages at the injury site at this early time. However, it is possible that microRNAs or regulatory proteins within the exosomes increase the production of anti-inflammatory cytokines or block M2 macrophages from converting to an M1 pro-inflammatory activation state. Future studies assessing the ratio of M2/M1 macrophages at later time points will be important to assess the effects of exosomes on macrophage population subtypes over time.

It is also unclear whether the actions of DiR-MSC^exos^ on spinal cord injury are due solely, or even primarily, to effects on macrophages. A very small number of DiR hotspots were also detected within astrocytic process endings (unpublished observation) and it is possible that exosomes were taken up by neurons or other cells as well, but at levels too low for us to detect. Hotspots of DiR fluorescence observed in this study almost certainly represent clusters of exosomes, since the sizes of individual exosomes (average 80 nm) are below the detection threshold for light microscopy. However, it is not known how many exosomes are required to produce a change in cell behavior and it is possible that cells may be influenced by DiR-MSC^exos^ uptake even if the numbers of exosomes within the cell are too small to produce a detectable DiR hotspot. Previous studies have identified several cell types in the CNS as potential targets for MSC^exos^. Other investigators have reported that intraventricularly delivered PKH26 labeled MSC^exos^ localized predominantly to astrocytes in diabetic mouse brains, but that some MSC^exos^ were also associated with neurons and microglia [56]. Likewise, uptake of PKH26 labeled MSC exosomes was detected within both neurons and microglia in mouse models of status epilepticus [35]. In addition, MSC-derived exosomes have been shown to enhance neurite outgrowth [38] and tube formation of endothelial cells [28] *in vitro*. These finding imply that MSC^exos^ may have multiple cell targets in the CNS, and that the M2 macrophage population may simply be the most easily identifiable target in this injury model.

While the trafficking IV DiR-MSC^exos^ to cells at the injury site supports the possibility of direct action of MSC^exos^ at the injury site, it is also possible that MSC^exos^ might influence SCI recovery by more indirect mechanisms. DiR hotspots were detected within the spleen over the same time course as their appearance at the contusion site, suggesting the possibility that MSC^exos^ might influence recovery by modulating overall immune system function. Several studies have shown that spinal cord injury in the rodent results in distinct changes in the immune system within 1-week post-SCI [64, 65]. These changes include a reduction in CD8^+^ lymphocytes and an increase in CD4^+^ lymphocytes in the spleen and lymph nodes and an increased proliferative response to myelin basic protein [64], as well as an overall immune suppression with a decrease numbers of circulating monocytes and lymphocytes in the blood [65]. The reduced weights of spleens in the SCI rats and the trafficking of DiR-MSC^exos^ to the spleen in this study support the idea that systemic immune responses to injury and MSC^exos^ infusion could play a role in SCI recovery. A recently published report [2] showing a decrease inflammatory and apoptotic markers in the contused spinal cord and improved motor recovery after intravenous infusion MSC-derived exosomes further supports the notion that MSC^exos^ modulate immune cell function within the injured spinal cord, but similarly cannot rule out a systemic immune system response, rather than a locally targeted action.

## Conclusions

The results of this study indicate that IV delivered MSC^exos^ rapidly traffic to the injured, but not the uninjured spinal cord, and associate specifically with M2 macrophages. MSC^exos^ were also detected in the spleen, which was notably reduced in weight in the SCI rat. Further studies will be needed to determine whether IV exosomes replicate the therapeutic actions of MSCs on SCI recovery as well as to elucidate the possible role of the spleen in SCI recovery.

## Supporting information

S1 Table Particle (exosome) sizes. (XLSX)

S2 Table MSC viability after DiR labelling. (XLSX)

S3 Table Distribution of "hotspots" within and outside of the spinal cord lesion. (XLSX)

S4 Table Cluster samples for NIS Elements analysis showing cluster lengths at 3 and 24 hr. (XLSX)

S5 Table Spleen weights for spinal cord injury (SCI)and age matched controls at 1 and 10 weeks. (XLSX)
